# Association between sex and outcomes in patients with non-small-cell lung cancer receiving combination chemoimmunotherapy as a first-line therapy: a systematic review and meta-analysis of randomized clinical trials

**DOI:** 10.1186/s40001-022-00789-7

**Published:** 2022-08-23

**Authors:** Kazuki Takada, Mototsugu Shimokawa, Fumitaka Mizuki, Shinkichi Takamori, Tomoyoshi Takenaka, Naoko Miura, Yasunori Shikada, Tomoharu Yoshizumi

**Affiliations:** 1 Department of Surgery, Saiseikai Fukuoka General Hospital, Fukuoka, Japan; 2 Department of Biostatistics, Yamaguchi University Graduate School of Medicine, 1-1-1 Minamikogushi, Ube, Yamaguchi, 755-8505 Japan; 3 Cancer Biostatistics Laboratory, Clinical Research Institute, National Hospital Organization Kyushu Cancer Center, 3-1-1 Notame, Minami-ku, Fukuoka, 811-1395 Japan; 4 Center for Clinical Research, Yamaguchi University Hospital, Yamaguchi, Japan; 5 Department of Surgery and Science, Graduate School of Medical Sciences, Kyushu University, Fukuoka, Japan

**Keywords:** Immune checkpoint inhibitor, Meta-analysis, Non-small-cell lung cancer, Sex

## Abstract

**Introduction:**

Recently, several meta-analyses have investigated the association between sex and the efficacy of immune checkpoint inhibitors (ICIs) in non-small-cell lung cancer (NSCLC). However, this issue remains controversial, because the results have been inconsistent. Moreover, the effect of sex on outcomes in patients with NSCLC receiving combination chemoimmunotherapy as a first-line therapy is poorly understood. The aim of this study was to examine the association between sex and outcomes in patients with NSCLC receiving combination chemoimmunotherapy as a first-line therapy.

**Methods:**

We searched PubMed and Scopus from database inception to Feb 18, 2022 and performed a systematic review and meta-analysis of randomized and controlled clinical trials investigating ICI+non-ICI vs non-ICI as a first-line therapy in NSCLC. The pooled hazard ratios (HRs) and 95% confidence intervals (CIs) for overall survival (OS) and progression-free survival (PFS) in male and female patients were calculated using common and random-effects models.

**Results:**

We analyzed 5,830 patients, comprising 4,137 (71.0%) males and 1,693 (29.0%) females, from nine randomized clinical trials. The pooled HR (95%CI) for OS comparing ICI+non-ICI vs non-ICI was 0.80 (0.72–0.87) for males and 0.69 (0.54–0.89) for females. The pooled HR (95%CI) for PFS comparing ICI+non-ICI vs non-ICI was 0.60 (0.55–0.66) for males and 0.56 (0.44–0.70) for females.

**Conclusions:**

In patients with NSCLC receiving combination chemoimmunotherapy as a first-line therapy, a greater improvement in OS and PFS was observed in female patients than in male patients.

## Introduction

Immune checkpoint inhibitors (ICIs), including anti-programmed cell death-1 (PD-1)/programmed death-ligand 1 (PD-L1) drugs (such as nivolumab, pembrolizumab, and atezolizumab) and anti-cytotoxic T-lymphocyte antigen-4 (CTLA-4) agents (ipilimumab), have become key treatments for patients with advanced or recurrent non-small-cell lung cancer (NSCLC) [25]. In addition, most patients with advanced NSCLC receive ICIs as a first-line combination chemoimmunotherapy in clinical settings worldwide [17].

Many recent studies, including meta-analyses, have reported the association between sex and the efficacy of ICIs in NSCLC based on the following observations [2–4, 12, 19, 23, 24, 26, 29–31] (1) Faster clearance of pathogens and greater vaccine effectiveness are observed in females compared with males [11, 28] (2) Females have higher rates of autoimmune disorders compared with males [11, 28]. These findings indicate that females might exhibit greater immunologic responses to antigens than males, and there might be a difference in the efficacy of ICIs between females and males. However, this issue remains controversial, because the results of previous meta-analyses have been inconsistent. For example, Conforti et al. conducted a meta-analysis of randomized and controlled clinical trials evaluating sex-based differences in response to first-line ICI monotherapy in patients with NSCLC expressing high PD-L1 levels and showed that the pooled hazard ratio (HR) and 95% confidence interval (CI) for overall survival (OS) reported in males vs females was 0.71 (0.64–0.98), indicating a significantly greater effect for males [4]. However, Xue et al. reported that there was no statistical difference in OS and progression-free survival (PFS) between males and females in a meta-analysis [30]. The meta-analysis by Xue et al. included patients treated with ICI monotherapy and ICI combination therapy as a first-line, second-line, or higher line therapy, and the heterogeneity may have contributed to the controversial results.

From these findings, the effect of sex on outcomes in patients with NSCLC receiving combination chemoimmunotherapy as a first-line therapy is poorly understood. Because this might be an important matter for clinicians involved in treating patients with advanced NSCLC, we conducted this updated meta-analysis to investigate the association between sex and outcomes in patients with NSCLC receiving combination chemoimmunotherapy as a first-line therapy.

## Materials and methods

### Study design

We searched PubMed and Scopus databases in accordance with the PRISMA (Preferred Reporting Items for Systematic Reviews and Meta-Analysis) guideline [13] from inception to Feb 18, 2022, and this meta-analysis was based on the data from published phase II or III randomized clinical trials (RCTs) investigating ICI+non-ICI vs non-ICI as a first-line therapy in advanced or recurrent NSCLC. The main search keywords used in the search strategy were (1) non-small-cell lung cancer or NSCLC, (2) immune checkpoint inhibitor or nivolumab or pembrolizumab or atezolizumab or avelumab or durvalumab or ipilimumab or tremelimumab or cemiplimab, and (3) study or trial. The inclusion criteria were defined according to the Population, Intervention, Comparison, Outcome, and Study design (PICOS) framework: (1) population: patients with NSCLC receiving treatment as a first-line therapy, (2) intervention: ICI + non-ICI, (3) comparison: non-ICI, (4) outcome: data available on HRs for OS or PFS in the overall population and sex subgroups, and (5) study design: RCTs. All duplicated clinical trials and single-arm phase I or II trials were excluded.

### Data extraction

Two authors (K.T. and F.M.) independently reviewed and extracted the following data from published papers: first author, journal name, year of publication, study ID and name, sample size according to sex and histology, drugs in the experimental and control arms, median follow-up time, and HRs for OS or PFS in the overall population and sex subgroups. Any disagreements were resolved through discussion and consensus between the two authors (K.T. and F.M.). The primary and secondary outcomes in this study were the pooled HRs and 95%CIs for OS and PFS in male and female patients calculated using common and random-effects models. The quality assessment of clinical trials included in this study was conducted using the Jadad scale [9].

### Statistical analysis

We conducted statistical analyses, generated forest plots, and detected publication bias in this meta-analysis using R software (version 3.4.0). The pooled HRs and 95%CIs for OS and PFS were calculated in male and female patients using common and random-effects models. All *P* values were two-sided, and *P* < 0.05 was considered statistically significant. Heterogeneity among studies was examined using *I*^2^ statistics, and it was considered low, moderate, and high for *I*^2^ values < 25%, 25–50%, and > 50%, respectively [6]. Publication bias was assessed using the funnel plot and Egger’s regression line.

## Results

### Published literature search and patients’ characteristics

**Fig. 1:**
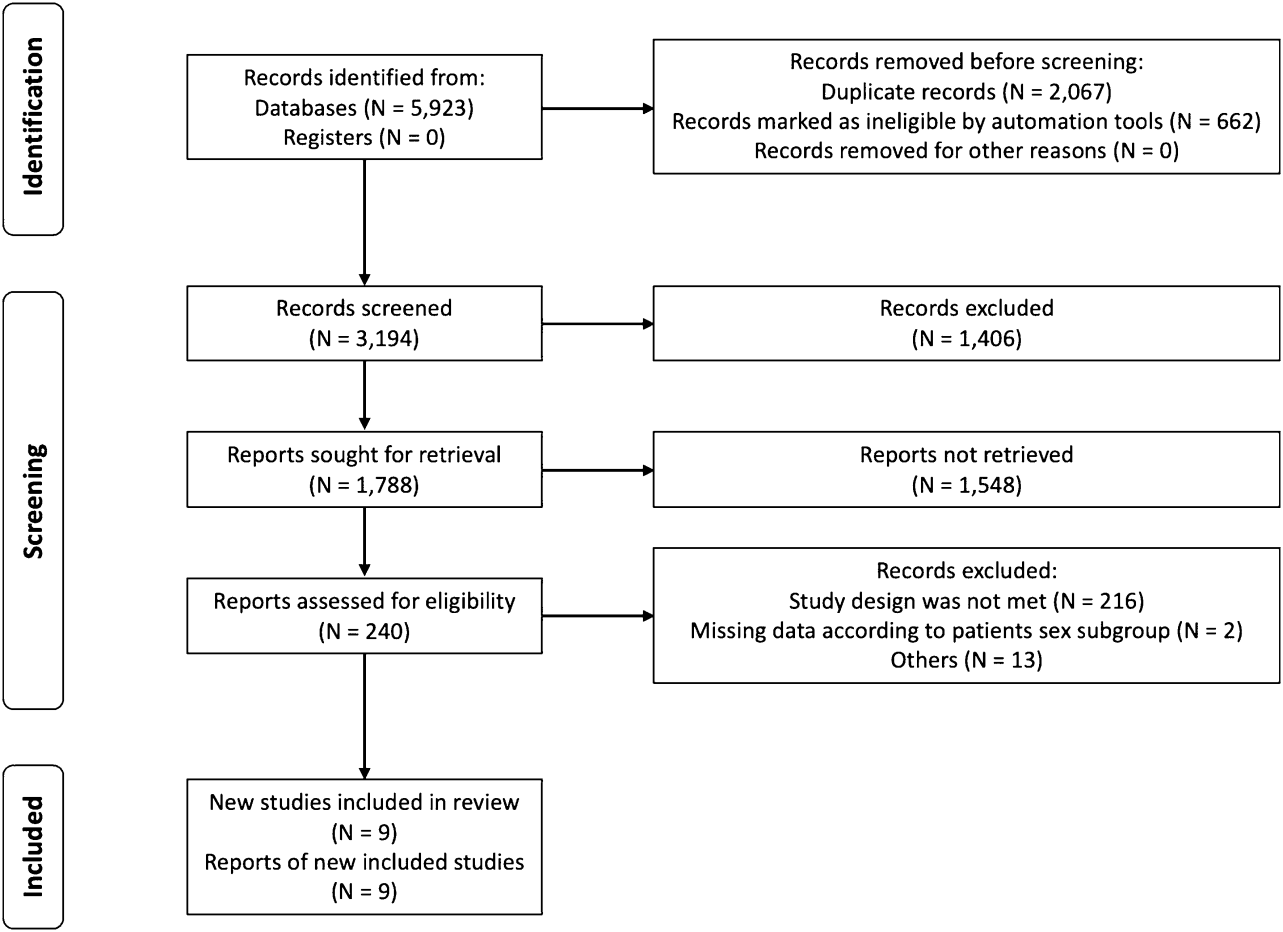
Flow diagram of the search process in this meta-analysis

First, we identified a total of 5,923 potentially relevant articles from PubMed and Scopus online databases using an initial search strategy. After screening and reviewing the titles, abstracts, and full texts, we finally included nine RCTs involving 5,830 patients in this study. Figure 1 shows the flow diagram of the search process.

The patients’ characteristics in the nine RCTs are listed in Table 1 [7, 10, 14–16, 18, 21, 22, 27]. Regarding the trials IMpower130 and IMpower150, we used the data in the intention-to-treat wild-type population, because these studies included patients with non-squamous NSCLC harboring epidermal growth factor receptor or anaplastic lymphoma kinase alterations [21, 27]. Among the 5,830 patients, 4,137 (71.0%) were male and 1,693 (29.0%) were female. There were five trials with data on both OS and PFS, three with only OS data, and one with only PFS data. The regimens of the experimental arm were as follows: PD-1 inhibitor (nivolumab or pembrolizumab) plus chemotherapy (*N* = 3), PD-L1 inhibitor (atezolizumab) plus chemotherapy (*N* = 4), CTLA-4 inhibitor (ipilimumab) plus chemotherapy (*N* = 1), and PD-1 inhibitor plus CTLA-4 inhibitor (nivolumab plus ipilimumab) plus chemotherapy (*N* = 1). All included RCTs were of high quality, with a score of 3 to 5 using the Jadad scale (Table 2).

**Table 1:** Features of randomized clinical trials included in the meta-analysis

Trial	Study ID	Sex, No		Histology, No		Experimental arm (No.)	Control arm (No.)	Median follow-up time	PFS HR (95%CI)			OS HR (95%CI)		
		Male	Female	Non-squamous	Squamous				Overall	Male	Female	Overall	Male	Female
NR	NCT01285609	635	114	0	749	Ipilimumab + chemotherapy (388)	Placebo + chemotherapy (361)	12.5 months vs 11.8 months	0.87 (0.75–1.01)	NA	NA	0.91 (0.77–1.07)	0.85 (0.71–1.02)	1.33 (0.84–2.11)
Govindan et al. J Clin Oncol 2017														
IMpower130	NCT02367781	400	279	679	0	Atezolizumab + chemotherapy (451)	Chemotherapy (228)	18.5 months vs 19.2 months	0.64 (0.54–0.77)	0.67 (0.54–0.85)	0.59 (0.45–0.78)	0.79 (0.64–0.98)	0.87 (0.66–1.15)	0.66 (0.46–0.93)
West et al. Lancet Oncol 2019														
IMpower131	NCT02367794	557	126	0	683	Atezolizumab + chemotherapy (343)	Chemotherapy (340)	26.8 months vs 24.8 months	NA	NA	NA	0.88 (0.73–1.05)	0.91 (0.75–1.12)	0.68 (0.44–1.04)
Jotte et al. J Thorac Oncol 2020														
IMpower132	NCT02657434	384	194	578	0	Atezolizumab + chemotherapy (292)	Chemotherapy (286)	28.4 months	0.56 (0.47–0.67)	0.61 (0.50–0.76)	0.48 (0.35–0.66)	0.86 (0.71–1.06)	0.93 (0.73–1.18)	0.76 (0.54–1.09)
Nishio et al. J Thorac Oncol 2021														
IMpower150	NCT02366143	428	269	697	0	Atezolizumab + bevacizumab + chemotherapy (359)	Bevacizumab + chemotherapy (338)	39.8 months vs 40.0 months	0.57 (0.48–0.67)	NA	NA	0.80 (0.67–0.95)	0.72 (0.58–0.90)	0.92 (0.70–1.22)
Socinski et al. J Thorac Oncol 2021														
KEYNOTE-189	NCT02578680	363	253	616	0	Pembrolizumab + chemotherapy (410)	Placebo + chemotherapy (206)	31.0 months	0.49 (0.41–0.59)	0.58 (0.46–0.74)	0.39 (0.29–0.52)	0.56 (0.46–0.69)	0.74 (0.56–0.96)	0.41 (0.30–0.56)
Rodríguez-Abreu et al. Ann Oncol 2021														
KEYNOTE-407	NCT02775435	455	104	0	559	Pembrolizumab + chemotherapy (278)	Placebo + chemotherapy (281)	7.8 months	0.56 (0.45–0.70)	0.58 (0.46–0.73)	0.49 (0.30–0.81)	0.64 (0.49–0.85)	0.69 (0.51–0.94)	0.42 (0.22–0.81)
Paz-Ares et al. N Engl J Med 2018														
TASUKI-52	NCT03117049	411	139	550	0	Nivolumab + Bevacizumab + chemotherapy (275)	Placebo + Bevacizumab + chemotherapy (275)	13.7 months	0.57 (0.46–0.72)	0.53 (0.41–0.69)	0.72 (0.45–1.15)	0.85 (0.63–1.14)	NA	NA
Sugawara et al. Ann Oncol 2021														
CheckMate 9LA	NCT03215706	504	215	495	224	Nivolumab + ipilimumab + chemotherapy (361)	Chemotherapy (358)	13.2 months	0.68 (0.57–0.82)	0.64 (0.52–0.79)	0.82 (0.60–1.14)	0.66 (0.55–0.80)	0.66 (0.53–0.82)	0.68 (0.47–1.00)
Paz-Ares et al. Lancet Oncol 2021														

*CI* confidence interval, *HR* hazard ratio, *NA* not available, *NR* not reported, *OS* overall survival, *PFS* progression-free survival

**Table 2:** Jadad Score of randomized clinical trials included in the meta-analysis

Trial	Study ID	Randomization	Randomization appropriate	Double-blind	Blinding appropriate	Description of withdrawals and dropouts	Total score
NR	NCT01285609	1	1	1	1	1	5
Govindan et al. J Clin Oncol 2017							
IMpower130	NCT02367781	1	1	0	0	1	3
West et al. Lancet Oncol 2019							
IMpower131	NCT02367794	1	1	0	0	1	3
Jotte et al. J Thorac Oncol 2020							
IMpower132	NCT02657434	1	1	0	0	1	3
Nishio et al. J Thorac Oncol 2021							
IMpower150	NCT02366143	1	1	0	0	1	3
Socinski et al. J Thorac Oncol 2021							
KEYNOTE-189	NCT02578680	1	1	1	1	1	5
Rodríguez-Abreu et al. Ann Oncol 2021							
KEYNOTE-407	NCT02775435	1	1	1	1	1	5
Paz-Ares et al. N Engl J Med 2018							
TASUKI-52	NCT03117049	1	1	1	1	1	5
Sugawara et al. Ann Oncol 2021							
CheckMate 9LA	NCT03215706	1	1	0	0	1	3
Paz-Ares et al. Lancet Oncol 2021							

*NR* not reported

### Effect of sex on OS

**Fig. 2:**
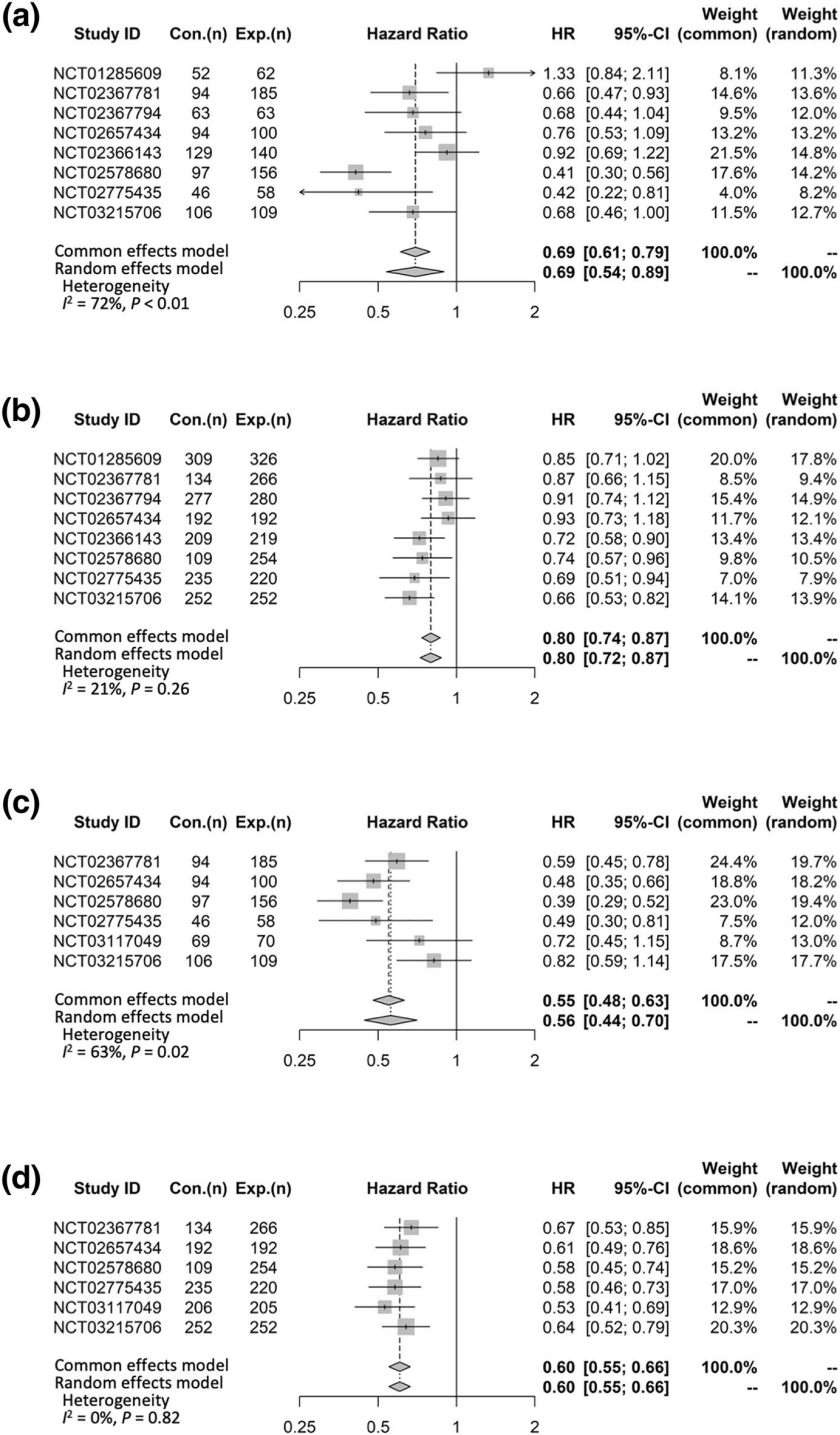
Forest plots of hazard ratios according to the patients’ sex. (a) OS in female patients. (b) OS in male patients. (c) PFS in female patients. (d) PFS in male patients. CI, confidence interval; Con., control arm; Exp., experimental arm; HR, hazard ratio; OS, overall survival; PFS, progression-free survival

Eight RCTs compared OS data on the basis of the patients’ sex. The pooled HR (95%CI) for OS comparing ICI + non-ICI vs non-ICI was 0.69 (0.54–0.89) for females and 0.80 (0.72–0.87) for males (Fig. 2a, b). There was between-study heterogeneity in females (*I*^2^ = 72%, *P* < 0.01) but not in males (*I*^2^ = 21%, *P* = 0.26) (Fig. 2a, b).

### Effect of sex on PFS

Six RCTs compared PFS data on the basis of the patients’ sex. The pooled HR (95%CI) for PFS comparing ICI + non-ICI vs non-ICI was 0.56 (0.44–0.70) for females and 0.60 (0.55–0.66) for males (Fig. 2c, d). There was between-study heterogeneity in females (*I*^2^ = 63%, *P* = 0.02) but not in males (*I*^2^ = 0%, *P* = 0.82) (Fig. 2c, d).

### Assessment of publication bias

**Fig. 3:**
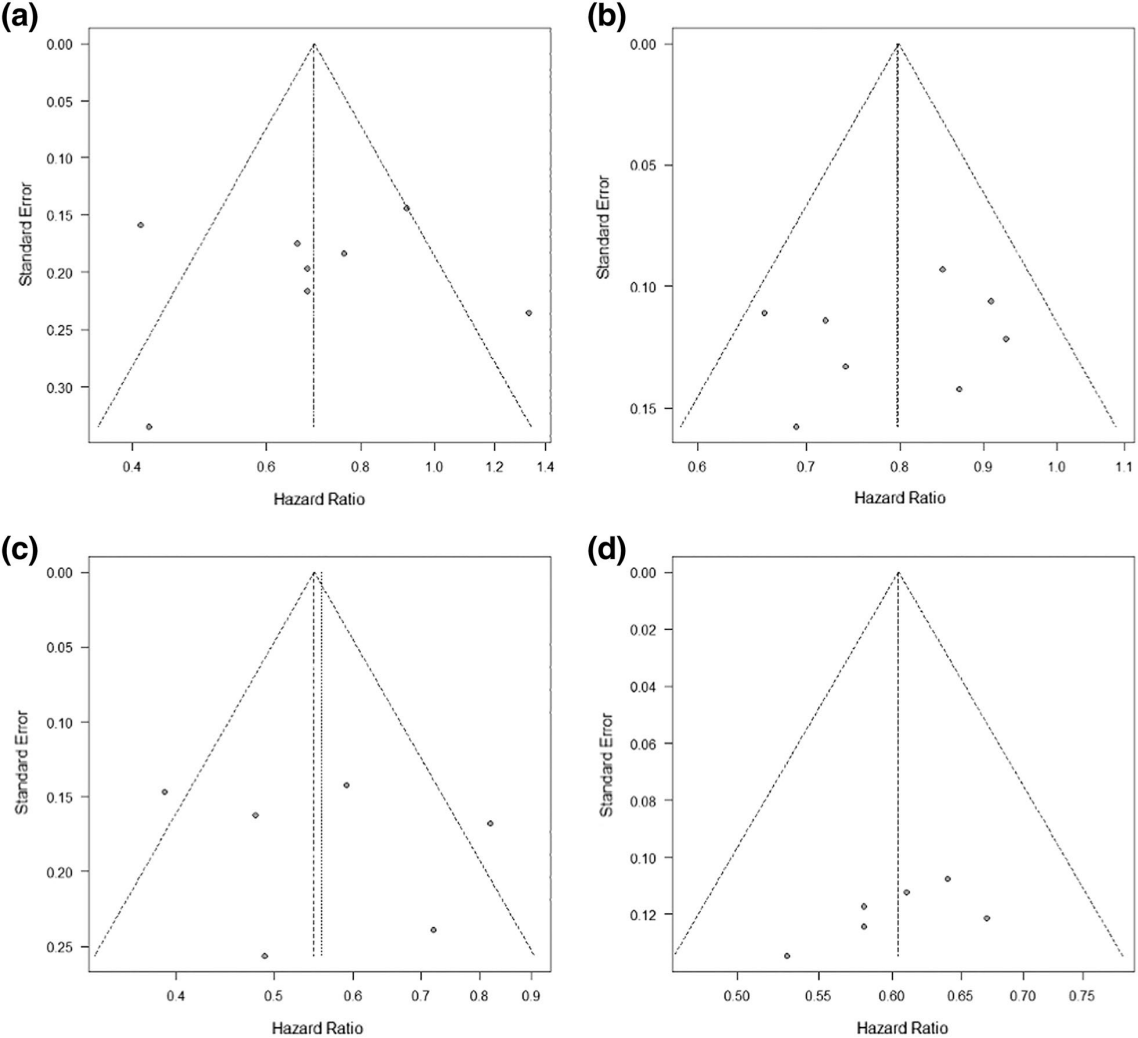
Funnel plot and Egger’s regression line. (a) OS in female patients. (b) OS in male patients. (c) PFS in female patients. (d) PFS in male patients. OS, overall survival; PFS, progression-free survival

We did not detect a high level of publication bias for OS and PFS in RCTs included in this study by the funnel plot and Egger’s regression line, as shown in Fig. 3.

## Discussion

This meta-analysis showed that the pooled HR (95%CI) for OS comparing ICI + non-ICI vs non-ICI was 0.69 (0.54–0.89) for females and 0.80 (0.72–0.87) for males, and the pooled HR (95%CI) for PFS comparing ICI + non-ICI vs non-ICI was 0.56 (0.44–0.70) for females and 0.60 (0.55–0.66) for males. These data indicate a greater improvement in OS and PFS in females than in males with NSCLC receiving combination chemoimmunotherapy as a first-line therapy. The results of our meta-analysis were similar to those of the previous meta-analysis by Conforti and colleagues. In their study, the pooled HR (95%CI) for OS comparing anti-PD-1/PD-L1 + chemotherapy vs chemotherapy was 0.48 (0.35–0.67) for females and 0.76 (0.66–0.87) for males, and the pooled HR (95%CI) for PFS comparing anti-PD-1/PD-L1 + chemotherapy vs chemotherapy was 0.56 (0.49–0.65) for females and 0.64 (0.58–0.71) for males [3]. Our meta-analysis included RCTs investigating the anti-CTLA-4 drug ipilimumab and used the most recent data. However, a recent study using individual-level data to examine the efficacy of chemoimmunotherapy compared with chemotherapy alone in advanced NSCLC patients revealed that the HR (95%CI) for OS was 0.83 (0.76–0.90) for females and 0.80 (0.74–0.87) for males, and these values were consistent in the propensity-score matched analysis with 0.88 (0.79–0.99) for females and 0.80 (0.72–0.88) for males [23]. The results of the above study by Tuminello et al*.* were inconsistent with those of the meta-analyses by Conforti and colleagues and our group. The meta-analyses were based on the results of RCTs, and patients included in RCTs have different clinical characteristics than patients in real-world settings. However, even the propensity-score matched analysis in the study by Tuminello et al*.* showed different results compared with those reported by Conforti et al*.* and our study.

Considering this issue, understanding the differences in the tumor microenvironment between females and males is important. Recently, Han et al. performed a comprehensive analysis to examine sex-based differences in tumor microenvironment-related characteristics in various cancers, including NSCLC [8]. Their study focused on the differences in tumor mutation burden, tumor microenvironment parameters (immune scores, stromal scores, tumor purity, and immune cells), immune checkpoint-related genes, and functional pathways [8]. For example, males had higher tumor mutation burdens than females among patients with lung adenocarcinoma, whereas females had higher immune scores than males among patients with both lung adenocarcinoma and lung squamous cell carcinoma. Furthermore, stromal scores were higher in female patients than in male patients with lung adenocarcinoma [8]. The immune and stromal scores of tumor tissues reflected the level of infiltrating immune and stromal cells. In addition, the above data indicated that female patients with lung adenocarcinoma and lung squamous cell carcinoma showed more infiltrating immune cells than male patients and that female patients with lung adenocarcinoma showed more infiltrating stromal cells than male patients. Overall, they concluded that both lung adenocarcinoma and squamous cell carcinoma showed the most significant sex biases (female-biased) in immune cells, immune checkpoint gene expression, and functional pathways, and they were classified into the ‘strong sex-biased’ immune group [8]. On the basis of these findings, we think that female patients experience improved survival with cancer immunotherapy compared with male patients, although the cancer immune system is extremely complex. Further investigations with a larger sample size on the association between sex and the efficacy of chemoimmunotherapy as a first-line treatment in advanced NSCLC are warranted.

There were several limitations associated with this study. First, this meta-analysis was based on the results of published RCTs and did not use patient-level data. Therefore, we could not directly compare benefits from cancer immunotherapy in addition to chemotherapy in female patients vs male patients. However, as mentioned above, this meta-analysis used the updated data in RCTs, including trials with not only PD-1/PL-L1 inhibitors but also CTLA-4 inhibitors (such as the CheckMate 9LA trial). To the best of our knowledge, this is the first meta-analysis of RCTs, including the CheckMate 9LA trial, to investigate the effect of sex on outcomes only in patients with NSCLC receiving combination chemoimmunotherapy as a first-line therapy. Moreover, the data used in this study (the results of the subgroup analyses in RCTs) were from pre-planned analyses, not ad-hoc analyses, and they were reliable. Second, the sample size was relatively small, especially for female patients. This may have prevented us from obtaining statistically accurate results. Third, some clinical trials included in this meta-analysis did not have adequate data, such as the event number in the experimental arm and control arm. Further validation in prospective future clinical trials might be required. Fourth, the protocol of this systematic review and meta-analysis was not registered in the Proportion of Systematic Review Protocols Registered Outside of the International Prospective Register of Systematic Reviews (PROSPERO) database. It is recommended that the protocol of systematic reviews and meta-analyses is registered in the PROSPERO database to avoid duplication and reduce reporting bias [1]. Therefore, the existence of reporting bias cannot be completely denied.

In addition to predicting the therapeutic response, sex and sexual activity can be risk factors for cancers [5, 20]. For example, Crocetto et al. concluded that sexual behaviors appeared to play a significant role in prostate cancer pathogenesis, whereas a correlation between sexual activity and testicular cancer had not yet been demonstrated, although the association between NSCLC and sexual activity remains unclear [5]. Therefore, sex is an important factor in cancer treatment.

In conclusion, patients with advanced NSCLC showed better OS and PFS with combination chemoimmunotherapy than chemotherapy alone as a first-line therapy regardless of sex, and a greater improvement in OS and PFS was observed in female patients than in male patients. Sex-related differences in response to combination chemoimmunotherapy should be taken into account when treating patients with advanced NSCLC.

## Data Availability

All the data used or generated during this study are available from the corresponding author on reasonable request.

## References

[1] Booth A, Clarke M, Dooley G, Ghersi D, Moher D, Petticrew M, Stewart L (2012) The nuts and bolts of PROSPERO: an international prospective register of systematic reviews Syst Rev 1:2 10.1186/2046-4053-1-2 10.1186/2046-4053-1-2 PMC3348673 22587842

[2] Conforti F (2018). Cancer immunotherapy efficacy and patients' sex: a systematic review and meta-analysis. Lancet Oncol.

[3] Conforti F (2019). Sex-based heterogeneity in response to lung cancer immunotherapy: a systematic review and meta-analysis. J Natl Cancer Inst.

[4] Conforti F (2021). Sex-based differences in response to anti-PD-1 or PD-L1 treatment in patients with non-small-cell lung cancer expressing high PD-L1 levels a systematic review and meta-analysis of randomized clinical trials. ESMO open.

[5] Crocetto F (2021). Impact of sexual activity on the risk of male genital tumors: a systematic review of the literature. Int J Environ Res Public Health.

[6] DerSimonian R, Laird N (1986). Meta-analysis in clinical trials. Control Clin Trials.

[7] Govindan R (2017). Phase III trial of ipilimumab combined with paclitaxel and carboplatin in advanced squamous non-small-cell lung cancer. J Clin Oncol.

[8] Han J (2022). Pan-cancer analysis reveals sex-specific signatures in the tumor microenvironment. Mol Oncol.

[9] Jadad AR, Moore RA, Carroll D, Jenkinson C, Reynolds DJ, Gavaghan DJ, McQuay HJ (1996). Assessing the quality of reports of randomized clinical trials: is blinding necessary?. Control Clin Trials.

[10] Jotte R (2020). Atezolizumab in combination with carboplatin and nab-paclitaxel in advanced squamous NSCLC (IMpower131): results from a randomized phase III. J Thoracic Oncol.

[11] Klein SL, Flanagan KL (2016). Sex differences in immune responses. Nat Rev Immunol.

[12] Lang D (2021). Sex-based clinical outcome in advanced NSCLC patients undergoing PD-1/PD-L1 inhibitor therapy-a retrospective bi-centric cohort study. Cancers.

[13] Liberati A (2009). The PRISMA statement for reporting systematic reviews and meta-analyses of studies that evaluate health care interventions: explanation and elaboration. PLoS Med.

[14] Nishio M (2021). Atezolizumab plus chemotherapy for first-line treatment of nonsquamous NSCLC: results from the randomized phase 3 IMpower132 Trial. J Thoracic Oncol.

[15] Paz-Ares L (2021). First-line nivolumab plus ipilimumab combined with two cycles of chemotherapy in patients with non-small-cell lung cancer (CheckMate 9LA): an international, randomised, open-label, phase 3 trial. Lancet Oncol.

[16] Paz-Ares L (2018). Pembrolizumab plus chemotherapy for squamous non-small-cell lung cancer. N Engl J Med.

[17] Reck M, Remon J, Hellmann MD (2022). First-line immunotherapy for non-small-cell lung cancer journal of clinical oncology : official journal of the American society of. Clin Oncol.

[18] Rodríguez-Abreu D (2021). Pemetrexed plus platinum with or without pembrolizumab in patients with previously untreated metastatic nonsquamous NSCLC: protocol-specified final analysis from KEYNOTE-189: official journal of the European society for medical oncology. Annals Oncol.

[19] Santoni M (2022). The impact of gender on The efficacy of immune checkpoint inhibitors in cancer patients: The MOUSEION-01 study. Critic Rev Oncol/Hematol.

[20] Schoentgen N (2021). Is it worth starting sexual rehabilitation before radical prostatectomy? results from a systematic review of the literature. Front Surg.

[21] Socinski MA (2021). IMpower150 Final Overall Survival Analyses for Atezolizumab Plus Bevacizumab and Chemotherapy in First-Line Metastatic Nonsquamous NSCLC. J Thorac Oncol.

[22] Sugawara S (2021). Nivolumab with carboplatin, paclitaxel, and bevacizumab for first-line treatment of advanced nonsquamous non-small-cell lung cancer. Annals Oncol.

[23] Tuminello S, Alpert N, Veluswamy RR, Kumar A, Gomez JE, Flores R, Taioli E (2022). Modulation of chemoimmunotherapy efficacy in non-small cell lung cancer by sex and histology: a real-world, patient-level analysis. BMC Cancer.

[24] Wallis CJD (2019). Association of patient sex with efficacy of immune checkpoint inhibitors and overall survival in advanced cancers: a systematic review and meta-analysis. JAMA Oncol.

[25] Wang C (2021). The landscape of immune checkpoint inhibitor therapy in advanced lung cancer. BMC Cancer.

[26] Wang C (2019). Effect of sex on the efficacy of patients receiving immune checkpoint inhibitors in advanced non-small cell lung cancer. Cancer Med.

[27] West H (2019). atezolizumab in combination with carboplatin plus nab-paclitaxel chemotherapy compared with chemotherapy alone as first-line treatment for metastatic non-squamous non-small-cell lung cancer (IMpower130): a multicentre, randomised, open-label, phase 3 trial. Lancet Oncol.

[28] Whitacre CC, Reingold SC, O'Looney PA (1999). A gender gap in autoimmunity. Science.

[29] Wu Y, Ju Q, Jia K, Yu J, Shi H, Wu H, Jiang M (2018). Correlation between sex and efficacy of immune checkpoint inhibitors (PD-1 and CTLA-4 inhibitors). Int J Cancer.

[30] Xue C (2021). Association between efficacy of immune checkpoint inhibitors and sex: an updated meta-analysis on 21 trials and 12,675 non-small cell lung cancer patients. Front Oncol.

[31] Yang F (2020). Association of sex age, and eastern cooperative oncology group performance status with survival benefit of cancer immunotherapy in randomized clinical trials: a systematic review and meta-analysis. JAMA Netw Open.

